# Chromatin signatures at transcriptional start sites separate two equally populated yet distinct classes of intergenic long noncoding RNAs

**DOI:** 10.1186/gb-2013-14-11-r131

**Published:** 2013-11-29

**Authors:** Ana C Marques, Jim Hughes, Bryony Graham, Monika S Kowalczyk, Doug R Higgs, Chris P Ponting

**Affiliations:** 1 MRC Functional Genomics Unit, Department of Physiology, Anatomy and Genetics, University of Oxford, South Parks Road, Oxford OX1 3QX, UK; 2 Department of Physiology, Anatomy and Genetics, University of Oxford, South Parks Road, Oxford OX1 3QX, UK; 3 MRC Molecular Haematology Unit, Weatherall Institute of Molecular Medicine, Oxford University, Oxford OX3 9DS, UK; 4 Current address: The Broad Institute of MIT and Harvard, Cambridge MA 02142, Massachusetts, USA

## Abstract

**Background:**

Mammalian transcriptomes contain thousands of long noncoding RNAs (lncRNAs). Some lncRNAs originate from intragenic enhancers which, when active, behave as alternative promoters producing transcripts that are processed using the canonical signals of their host gene. We have followed up this observation by analyzing intergenic lncRNAs to determine the extent to which they might also originate from intergenic enhancers.

**Results:**

We integrated high-resolution maps of transcriptional initiation and transcription to annotate a conservative set of intergenic lncRNAs expressed in mouse erythroblasts. We subclassified intergenic lncRNAs according to chromatin status at transcriptional initiation regions, defined by relative levels of histone H3K4 mono- and trimethylation. These transcripts are almost evenly divided between those arising from enhancer-associated (elncRNA) or promoter-associated (plncRNA) elements. These two classes of 5′ capped and polyadenylated RNA transcripts are indistinguishable with regard to their length, number of exons or transcriptional orientation relative to their closest neighboring gene. Nevertheless, elncRNAs are more tissue-restricted, less highly expressed and less well conserved during evolution. Of considerable interest, we found that expression of elncRNAs, but not plncRNAs, is associated with enhanced expression of neighboring protein-coding genes during erythropoiesis.

**Conclusions:**

We have determined globally the sites of initiation of intergenic lncRNAs in erythroid cells, allowing us to distinguish two similarly abundant classes of transcripts. Different correlations between the levels of elncRNAs, plncRNAs and expression of neighboring genes suggest that functional lncRNAs from the two classes may play contrasting roles in regulating the transcript abundance of local or distal loci.

## Background

Eukaryotic genomes are pervasively transcribed [1,2] with evidence for up to three-quarters of nucleotides in the human genome being expressed in at least one cell type during development [2]. Transcripts lacking an apparent open reading frame are often classified simply based on their length, the absence of protein-coding potential and their location in the genome relative to protein-coding genes [3,4]. An intriguing class of noncoding transcripts are those exceeding 200 nucleotides in length and transcribed from loci that are intergenic relative to protein-coding genes (intergenic long noncoding RNAs (lncRNAs)). At least 50,000 lncRNAs are expressed from intergenic regions of the human genome, more than twice the number of protein-coding genes [5]. Compared to protein-coding transcripts, intergenic lncRNAs are generally less abundant and their expression is more spatially and temporally restricted [4,6]. Genome-wide analysis of mammalian intergenic lncRNA sequence [7,8] and transcription [9,10] has revealed that, in general, these loci have been conserved during evolution, albeit at substantially lower levels than protein-coding genes, suggesting that at least some intergenic lncRNAs may have conserved biological roles. Biological functions attributed to the handful of well-characterized intergenic lncRNAs are diverse, ranging from transcriptional control to post-transcriptional modulation of gene expression (for recent reviews see [11-13]).

In this study, for simplicity, we refer to intergenic lncRNAs as those that are transcribed by RNA-polymerase II, 5′ end capped and polyadenylated. Here we address two important, and incompletely answered, questions concerning the origins (transcriptional initiation regions (TIRs)) and classification of intergenic lncRNAs. First, what is the relative prevalence of promoter- and enhancer-associated transcripts within sets of transcripts that are annotated simply as being intergenic lncRNAs? Second, do differences in the chromatin status at intergenic lncRNA TIRs reflect their potential function?

Histone modifications allow the distinction between different types of regulatory elements [14,15]. Promoters of transcribed protein-coding genes, for example, are enriched in trimethylation of lysine 4 of histone H3 (H3K4me3) [14,15]. Some intergenic lncRNA loci have been defined previously using chromatin signatures that are similar to those often found at protein-coding genes, namely H3K4me3 marked promoters and trimethylation of lysine 36 of histone H3 (H3K36me3) across transcribed regions [16]. These findings demonstrate that some intergenic lncRNAs are transcribed from promoter-like elements.

A second class of transcripts could be prevalent in current catalogues of intergenic lncRNAs, namely enhancer-associated noncoding RNAs (eRNAs) [17]. Transcription is a common feature of active mammalian enhancers and can give rise to both non-polyadenylated, bidirectional, unstable transcripts [17] as well as unidirectionally transcribed, polyadenylated, relatively stable and sometimes spliced eRNAs [18,19]. We have previously shown that activation of enhancers located within protein-coding genes promotes transcription of long noncoding RNAs that utilize splicing and polyadenylation signals from their protein-coding hosts to produce stable unidirectional eRNAs [20]. On the other hand, the expression of intergenic lncRNA loci has been associated with enhanced levels of their neighboring protein-coding genes, both through genome-wide [10,21,22] and locus-specific analyses [22,23], suggesting that a large, yet undetermined, fraction of transcripts within lncRNA catalogues are unidirectional eRNAs, as previously proposed by Natoli and Andrau [24]. These observations motivated us to expand on our earlier observations [10,20] to determine to what extent intergenic lncRNAs might originate from active intergenic enhancers.

To address this question we generated new genome-wide maps of H3K4me3 and monomethylation of lysine 4 of histone H3 (H3K4me1 and H3K4me3, respectively), deep poly(A) + RNA sequencing and nanoCAGE [25,26] data from purified mouse erythroblasts. Using these data, we annotated a stringent set of intergenic lncRNAs expressed in these cells and accurately defined their transcriptional start sites using these newly acquired nanoCAGE data. We used the relative abundance of H3K4me1 and H3K4me3 at these intergenic lncRNAs’ TIRs, a well-established and widely used approach to differentiate between promoter and enhancer-like regulatory elements [27], to distinguish unidirectional eRNAs (here called elncRNAs) from promoter-associated lncRNAs (or plncRNAs). Our analyses demonstrate that chromatin marks at their TIRs effectively separate two equally prevalent classes of intergenic lncRNAs. These classes differ with respect to their evolution, tissue-specificity, levels of expression and co-expression levels with their neighboring genes, suggesting that, if they influence gene expression, they may do so in different ways.

## Results

### More than half of lncRNAs originate from enhancer-like regions

The functional *cis*-elements, *trans*-acting factors and epigenetic modifications associated with gene expression during the well-defined cellular stages of erythropoiesis have been studied extensively [28-30]. This molecular and cellular model is thus ideal for studying the causes and potential consequences of lncRNA transcription.

We used nanoCAGE [26] to capture and sequence the 5′ ends of purified mouse (C57BL/6 J) intermediate erythroblast expressed transcripts and to annotate their TIRs in these cells. We defined TIRs as previously [25] by clustering the 5′ end positions of reads mapping within 20 nucleotides on the same strand. Clusters closer than 400 nucleotides were then considered to be part of the same TIR (Materials and methods). Integrating nanoCAGE and paired-end transcription (RNA-Seq) data allowed us to identify 11,689 polyadenylated transcripts (Additional file 1) originating from 7,608 TIRs that overlap DNase 1 hypersensitive sites in mouse intermediate erythroblasts (Materials and methods; Additional files 2 and 3). As expected [31], the nanoCAGE read count supporting a given TIR correlates well (Pearson R = 0.44) with the expression level of its associated transcript (Additional file 4). Most (95.4%) transcripts overlap by one or more nucleotides a protein-coding gene annotation (ENSEMBL build 68 [32]) and for simplicity we refer to these as protein-coding transcripts. Of the remaining intergenic transcripts, 391 had no protein-coding potential [33] and were longer than 200 nucleotides and thus were annotated as being lncRNAs. A small, but significant (6; 36-fold enrichment, *P* < 1 × 10^-3^; Materials and methods), number of these lncRNAs were also identified as being expressed in erythroblasts in an earlier experiment [28]. Differences between the two catalogues are likely due to the use of different experimental methods (RNA hybridization or sequencing) and the conservative approach used in the current study to annotate intergenic lncRNA transcripts. Indeed, when we considered overlapping RNAseq reads to be sufficient evidence of expression, 44% (199) of the previously reported lncRNAs found to be expressed in mouse intermediate erythroblasts [28] were identified as expressed in our experiment.

**Figure 1:**
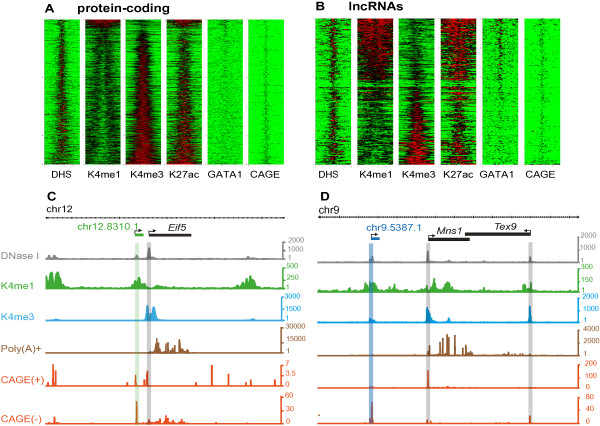
**Contrasting epigenetic landscapes at transcriptional start sites for protein-coding genes and lncRNAs in mouse intermediate erythroblasts.** The heatmap represents the distribution of DNAse I hypersensitive sites (DHS), H3K4me1, H3K4me3, H3K27ac, Gata1 and NanoCAGE signal (red high, green low) in a 4 kbp window centered around the middle of the nanoCAGE-defined TIRs for **(A)** protein-coding genes and **(B)** lncRNA loci. Representative novel **(C)** elncRNA (green, chr12:112754628 to 112804627 (mm9)) and **(D)** plncRNA (blue, chr9:72229000 to 72364999 (mm9)) were annotated *de novo* from C57BL/6 erythroid cell poly(A) + RNA (brown). Their transcriptional start sites were defined using strand-specific nanoCAGE (red; plus and minus signs represent density of reads within strand-specific libraries) found within DHS regions (grey). H3K4me1, green; H3K4me3, blue. Arrows on elncRNA and plncRNA and their neighboring transcript indicate the direction of transcription.

To classify TIRs associated with these annotations we used genome-wide chromatin immunoprecipitation followed by sequencing (ChIP-seq) to identify regions enriched in H3K4me1 or H3K4me3 (Materials and methods). A relatively high level of H3K4me1- over H3K4me3-modified chromatin is a well-established property of enhancers [20,34,35] that has been extensively used to generate genome-wide catalogues of enhancer- or promoter- like regulatory elements [36]. Here we generated DNase-Seq data to identify all active regions within the genome and then quantified these regions’ relative enrichment of H3K4me1 and H3K4me3. On the basis of their difference in these marks, active elements were then effectively sorted into two clearly demarcated classes (Additional file 5) that show characteristics of either promoters or enhancers [20]. In such a way TIRs were classified as either enhancer-like (H3K4me1^high^, 686 TIRs) or promoter-like (H3K4me3^high^, 6824 TIRs) (Additional file 6). As expected, these regions are enriched in acetylation of lysine 27 of histone 3 (H3K27ac; Figure 1; Additional file 5), a well-accepted mark of biological activity.

As expected, most (92%) protein-coding transcripts initiate at promoter-like TIRs (Figure 1A). The 568 protein-coding transcripts originating from enhancer-like TIRs (Figure 1A) substantially overlap our previously reported set [20] of 176 enhancer-associated alternative first exons identified in this cell-type (5.7-fold enrichment, permutation test *P* < 10^-3^). Of the top 15 enhancer-associated protein-coding transcripts, 12 correspond to enhancer-associated intragenic lncRNAs (meRNAs) identified in our previous study [20], including 2 meRNAs initiating at the α-globin enhancers (Additional file 7).

Next we considered the set of 391 TIRs associated with lncRNA expression. Consistent with an earlier report [37], more than a third of lncRNAs (152, 38%) are transcribed from bidirectional, protein-coding gene TIRs. The chromatin associated with most (97%) bidirectional TIRs is enriched with H3K4me3. These lncRNAs were not considered further in this analysis. Hereafter we refer to the 239 lncRNAs that are entirely intergenic (that is, they overlap neither protein-coding genes nor their TIRs) as intergenic lncRNAs. In contrast to protein-coding genes, more than half (124, 52%) of these lncRNAs originate at enhancer-like rather than at promoter-like TIRs (Figure 1B; Additional file 8). We refer to these transcripts as intergenic enhancer-associated lncRNAs or elncRNAs (Figure 1D). The remaining 115 intergenic lncRNAs arise from promoter-like TIRs, and hence we refer to these as intergenic promoter-associated lncRNAs or plncRNAs (Figure 1C; Additional file 9). It has previously been shown that many active erythroid enhancers are bound by the tissue-restricted transcription factor Gata1 [29,38]. Consistent with this, we found an enrichment of Gata1 binding at the TIRs of elncRNAs but not plncRNAs (Figure 1A,B).

### Comparisons of the sequence features of elncRNA and plncRNA transcripts

**Figure 2:**
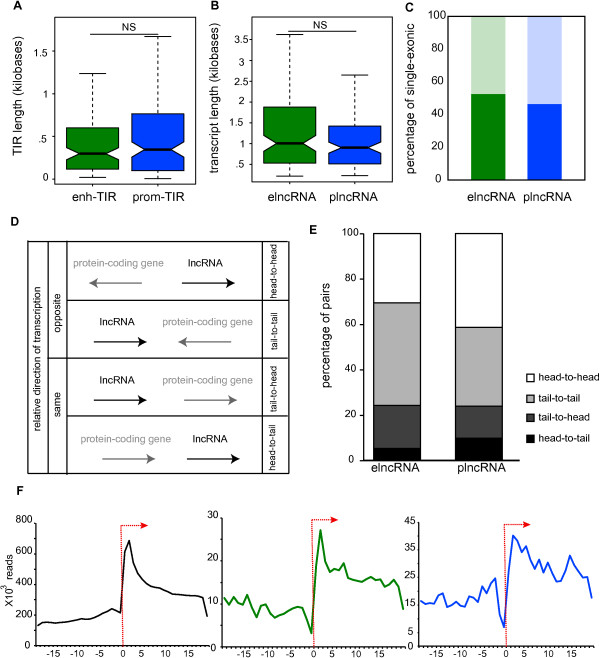
**elncRNA and plncRNA loci have similar sequence features. (A)** The lengths of elncRNA (enh-TIR, median = 293 nucleotide, green) and plncRNA (prom-TIR, median = 342 nucleotide, blue) TIRs are similar (two-tailed Mann–Whitney test, *P* = 0.52), **(B)** as are the lengths of elncRNA (median = 1006 nucleotide) and plncRNA (median = 903 nucleotide) transcripts (two-tailed Mann–Whitney test, *P* = 0.24). **(C)** The percentage of elncRNAs that are mono-exonic (47%, dark green) is similar to that for plncRNAs (54%, dark blue, two-tailed Fisher’s exact test, *P* = 0.37). Light green and blue represent the percentage of multiexonic elncRNAs and plncRNAs, respectively. **(D)** Relative transcriptional orientation for lncRNAs (black) and their closest protein-coding gene (grey). For simplicity lncRNAs are represented here as transcribed from 5′ to 3′. Arrow represents the direction of transcription. **(E)** Percentages of lncRNA-protein-coding gene pairs with relative orientation head-to-head (white; elncRNAs = 31%, plncRNAs = 41%); tail-to-tail (light-grey, elncRNAs = 45%, plncRNAs = 35%); tail-to-head (dark-grey; elncRNAs = 19%, plncRNAs = 14%) and head-to-tail (black; elncRNAs = 5%, plncRNAs = 10%). **(F)** Total number of polyA selected RNA sequencing reads (y-axis) associated with the transcriptional start sites of protein-coding gene (black), elncRNA (green) and plncRNA (blue) meta-genes’ transcriptional start sites (±50 bp, x-axis). Arrow indicates the location of the transcriptional start site and the direction of the meta-gene transcription. NS, not significant.

To address whether differences in histone marks at their origin reflect differences in the properties of elncRNAs and plncRNAs, we next investigated their sequence features. The TIR sizes and transcript lengths of both classes of lncRNAs (Figure 2A,B) are similar, as are the fractions of multiexonic transcripts within each class (Figure 2C). Therefore these sequence features do not permit the distinction of elncRNAs from plncRNAs.

We also determined the orientation of transcription of plncRNAs and elncRNAs relative to their closest protein-coding genes (Figure 2D). Both elncRNAs and plncRNAs are preferentially transcribed in the opposite direction to that of their nearest protein coding neighbors. We noted a non-significant trend for plncRNAs, relative to elncRNAs, and their closest protein-coding genes to lie in a head-to-head orientation (Figure 2E). Probably, as a consequence, the TIRs of plncRNAs and their protein-coding gene neighbors (median = 29.6 kb) are significantly closer to each other than are the TIRs of elncRNAs and their adjacent protein-coding genes (median = 44.1 kb) or are pairs of protein-coding genes (median = 48.5 kb; Additional file 10). Also, the density of poly(A)-selected reads mapped around elncRNA and plncRNA transcriptional start sites revealed that, in terms of directionality, the two classes of lncRNAs are predominantly unidirectional and indistinguishable from each other or protein-coding transcripts (Figure 2F). A similar analysis using poly(A)-depleted RNA sequencing reads revealed that their TIRs exhibit similar signatures to those previously described for promoter- and enhancer-like elements, as expected [2,17] (Additional file 11).

### elncRNAs and plncRNAs have different origins and different patterns of expression

**Figure 3:**
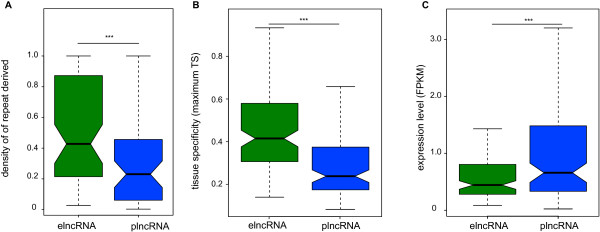
**elncRNA and plncRNA loci tend to show different origins and are associated with different transcriptional properties. (A)** Density of transposable element (TS)-derived sequence in elncRNA (green) and plncRNA (blue) exons. **(B)** elncRNAs (median (elncRNA_maxTS_) = 0.41) tend to be more tissue-specific (two-tailed Mann–Whitney test, *P* < 5 × 10^-11^) than are plnRNAs (median (plncRNA_maxTS_) = 0.22). **(C)** elncRNAs (median FPKM, (elncRNA_exp_) = 0.43) tend to be more lowly expressed in erythroblasts than are plncRNAs (median FPKM, (plncRNA_exp_) = 0.64, two-tailed Mann–Whitney test, *P* < 0.001). ***P < 0.001. FPKM, fragments per kilobase of sequence per million reads.

Next we investigated the DNA sequence underlying the TIRs of intergenic lncRNAs. It has previously been shown that transposable elements are enriched both at enhancer elements [39] and at the promoters of intergenic lncRNAs [40,41]. Here we find more specifically that the coverage of transposable element-derived TIRs by transposable element sequence is higher for elncRNAs (median density = 0.4, two-tailed Mann–Whitney test, *P* < 0.002) than for plncRNAs (median density = 0.23) loci (Figure 3A). Short interspersed nuclear elements (SINEs) are particularly highly associated with elncRNA TIRs (40% higher, permutation test, *P* < 0.01). Intergenic enhancers that are unidirectionally transcribed are rarely associated with CpG islands [19] and in agreement we found that none (0) of the annotated elncRNA TIRs are associated with CpG islands. By contrast, nearly half (48%, 66) of plncRNA TIRs overlap a CpG island (>1 nucleotide), a significantly higher fraction (*P* < 0.001, two-tailed Fisher’s exact test). The different origins of elncRNAs’ and plncRNAs’ TIRs suggest that these lncRNAs may have different patterns of expression and, if functional, may play different roles. During this manuscript’s preparation Schlesinger *et al*. [42] also proposed that CpG density might allow the distinction of transcribed promoter- from enhancer-like regions.

To determine how intergenic elncRNAs and plncRNAs are expressed throughout differentiation and development we used Cufflinks [43] to estimate their abundance (fragments per kilobase of sequence per million reads (FPKM)) in intermediate erythroblasts, in normal adult tissues and in cell lines for which data are publicly available [44] (Additional file 12). To estimate cell specificity, we identified the tissue in which each transcript is most highly expressed and then estimated the fold increase in expression in that tissue relative to its median expression across all tissues tested (maximum tissue specificity, max Ts). As observed previously [4,6], in intermediate erythroblasts, intergenic lncRNAs are, on average, two times more tissue specific and ten-fold less abundant than protein-coding transcripts (data not shown). The comparison between intergenic lncRNA classes revealed that elncRNAs tend to have a more restricted pattern of expression (Figure 3B) and are less abundant in intermediate erythroblasts than plncRNAs (Figure 3C). Consistent with their lower abundance and restricted expression, we found that 88% (110) of the elncRNAs identified here are novel (that is, they show no overlap with ENSEMBL lncRNA transcripts). This fraction is significantly higher than found for plncRNAs, of which only 56% are not annotated (65, two-tailed Fisher’s exact test, *P* < 0.001).

### elncRNA sequence is not constrained during mammalian evolution

**Figure 4:**
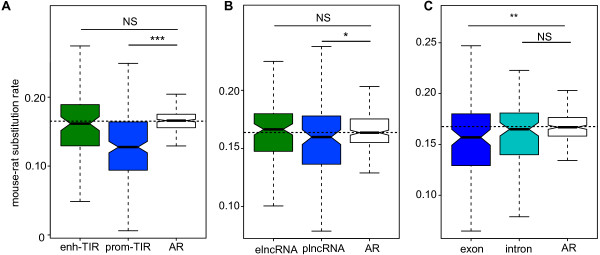
**plncRNA but not elncRNA loci are selectively constrained over rodent evolution. (A)** Mouse-rat nucleotide substitution rate for elncRNA TIRs (median d_elncRNA-TIR_ = 0.161, green) and plncRNA TIRs (median d_plncRNA-TIR_ = 0.127, blue), and for neighboring putatively neutrally evolving sequence (ancestral repeats (ARs), white). Horizontal dashed line represents the neutral expectation (median d_AR_ = 0.165). **(B)** Mouse-rat nucleotide substitution rate for elncRNA loci (d_elncRNA-loci_ = 0.166, green) and plncRNA loci (d_plncRNA-loci_ = 0.159, blue), and ARs (white). **(C)** Mouse-rat nucleotide substitution rate for transcribed and spliced plncRNA exons (d_plncRNA-exon_ = 0.156, violet) and plncRNA introns (d_plncRNA-intron_ = 0.165, turquoise), and ARs (white). Two-tailed Mann–Whitney test, ***P < 0.001; **P < 0.01; *P < 0.05; NS, not significant.

Evolutionary constraint should reflect conservation of function and therefore we assessed this for intergenic elncRNAs and plncRNAs by comparing the nucleotide substitution rates at their TIRs in rodents. We estimated these rates by comparing the substitutions in aligned mouse and rat TIR sequences with the corresponding rate at neighboring (presumed neutrally evolving) sequence with matching G + C content (Materials and methods, *d*_*neutral*_) [8]. The substitution rate of promoter-like TIRs was significantly lower (20% on average; two-tailed Mann–Whitney test, *P* < 1 × 10^-14^; Figure 4A) than the proxy neutral rate. By contrast, the substitution rate for enhancer-like TIRs was not significantly different from the neutral rate (two-tailed Mann–Whitney test, *P* = 0.35; Figure 4A).

Next we investigated the evolutionary signatures, in the two rodents, across the full length (exons and introns) of elncRNA or plncRNA loci. Consistent with the lack of detectable evolutionary constraint at their respective TIRs, we found that elncRNA loci accumulate substitutions at the same rate as neighboring neutrally evolving sequence (two-tailed Mann–Whitney test, *P* = 0.24; Figure 4B). By contrast, we found plncRNA loci to be selectively constrained (two-tailed Mann–Whitney test, *P* < 0.05; Figure 4B), accumulating on average 5% fewer substitutions per nucleotide than neutral sequence. This is consistent with negative selection purging a small number of deleterious mutations at these loci. To determine whether it is transcription across the loci or the plncRNA transcript *per se* that has been the subject of constraint during rodent evolution, we compared the substitution rates of plncRNA introns and exons. We found that plncRNA exons (two-tailed Mann–Whitney test, *P* < 0.01; Figure 4C) but not introns (two-tailed Mann–Whitney test, *P* = 0.19; Figure 4C) were selectively constrained. This suggests that when plncRNAs are functional, their functions can be conserved and will more often be transacted by mature rather than by unspliced transcripts than through the act of transcription *per se*. Neither elncRNA exons nor their introns exhibited non-neutral evolution (two-tailed Mann–Whitney test, *P* > 0.1; Additional file 13), further supporting the notion that elncRNA sequence is not constrained.

Similar results were obtained when substitution rates were estimated by aligning mouse to orthologous human sequence, rather than to rat sequence (Additional file 14). Consequently, the differences in constraint observed for plncRNAs and elncRNAs have persisted throughout mammalian evolution. In summary, our findings reveal that whilst plncRNA sequence and associated TIRs have evolved under evolutionary constraint no such evidence is detectable for intergenic elncRNAs or their TIRs.

### elncRNA expression is associated with enhanced levels of expression from neighboring protein-coding genes

The levels of expression of intergenic lncRNAs in general correlate with the levels of transcription from adjacent protein-coding genes [10,21,22,45]. Here we asked whether this correlation was derived from either one or both of the lncRNA classes defined in this study. elncRNA transcription was found to occur five times more frequently than expected by chance (Materials and methods; permutation test *P* < 0.005) in the vicinity of protein-coding genes whose expression is higher (five-fold) in intermediate erythroblasts than in any other tissue. By contrast, no such enrichment was found for plncRNA loci (2% depletion, permutation test *P* = 0.35). Equivalent results were obtained when the threshold of intermediate erythroblast relative to other tissues expression was set to 2- or 10-fold (data not shown).

To estimate the extent of the enhancement in protein-coding gene expression observed in association with neighboring lncRNA transcription we used publicly available transcriptome data from three stages of erythroid development to estimate the transcript levels of lncRNAs and protein-coding genes in progenitor (BFU-e), early erythroid (CFU-e) and intermediate erythroblast (Terr119+) cells [28] (Materials and methods). We considered only those transcripts whose expression could be detected in at least one of the three developmental stages (41% of elncRNAs, 53% plncRNAs, and 85% mRNAs). As previously (Figure 3B), the expression of elncRNAs tended to be restricted to fewer cell types (Additional file 15).

**Figure 5:**
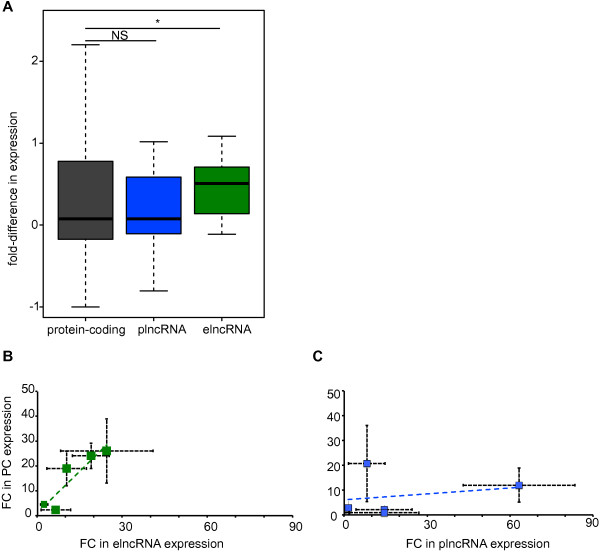
**Expression of elncRNA but not plncRNA loci is associated with enhanced expression levels of neighboring protein-coding genes. (A)** Fold difference, Δ, in expression of the neighboring loci in the stage in which the expression level of the reference locus (protein-coding gene, plncRNA or elncRNA) is maximal relative to the stage in which it is minimal. Δ is defined as (Expression_max_ - Expression_min_)/Expression_min_. Two-tailed Mann–Whitney test, *P < 0.05; NS, not significant. Fold-change (FC) between Ter119+ and colony-forming unit erythroblast (CFUE) expression (measured by quantitative PCR) of **(B)** elncRNAs (green) or **(C)** plncRNAs (blue) (x-axis) and their respective protein-coding gene (PC, y-axis). Data used for the preparation of this figure are available in Additional file 16. Horizontal and vertical bars represent the standard error of the mean for fold-change of lncRNA and protein-coding genes, respectively. Dashed blue and green lines represent the elncRNAs’ and plncRNAs’ linear regression functions, respectively.

We considered whether the abundance of elncRNAs and plncRNAs correlated with the levels of transcripts from adjacent protein-coding genes (Materials and methods). Pairs of lncRNA loci and their closest neighboring protein-coding genes that both showed detectable expression in at least one erythropoietic stage were identified [28] (Materials and methods; 17 elncRNAs and 28 plncRNAs), together with a control set of 9,770 similarly identified pairs of adjacent protein-coding genes. For each lncRNA in a pair we identified the two developmental stages in which its expression was either minimal or maximal, and calculated the expression fold difference, Δ, for its neighboring protein-coding gene at these two stages. If there was no correlation between the expression levels of adjacent genomic loci then Δ is expected to be distributed around zero. The distribution of Δ values for genes that are adjacent to plncRNA loci (median Δ = 0.075) was similar to Δ values for control pairs of adjacent protein-coding loci (median Δ = 0.076, two-tailed Mann–Whitney test, *P* = 0.97; Figure 5A). By contrast, despite being more distant, on average, from their neighboring protein-coding genes than are plncRNA loci (Additional file 3), Δ values for the protein-coding genes adjacent to elncRNA loci were, on average, 6.6-fold higher (median Δ = 0.50, two-tailed Mann–Whitney test, *P* < 0.05; Figure 5A).

This increased expression of protein-coding genes lying near to elncRNA loci may reflect regional changes in chromatin that affect both coding and noncoding loci [46] or could be due to a specific association between transcription of the elncRNA and the nearest protein-coding gene in its vicinity. To distinguish between these possibilities, we also calculated Δ for protein-coding genes lying adjacent to the nearest neighbor. We found that expression levels of these 'next-but-one’ genes were not correlated with elncRNA expression levels (Δ = -0.21, two-tailed Mann–Whitney test, *P* = 0.27). To validate the results obtained from this genome-wide analysis we used quantitative PCR (qPCR) to investigate the association in expression between 10 protein-encoding RNAs and their genomically proximal 5 plncRNA or 5 elncRNA loci (Figure 5B,C; Additional file 16). The correlation between the fold-change in expression of these elncRNA loci and their neighboring protein-coding genes (Pearson R = 0.82) is considerably higher than that for pairs of plncRNA loci and their adjacent protein-coding genes (Pearson R = 0.05), consistent with our genome-wide observation.

We conclude that while the restricted spatial and temporal expression of elncRNAs is associated with enhanced expression of its neighbors, no such correlation can be found for the broadly expressed plncRNA loci.

## Discussion

We recently found that that some intragenic lncRNAs originate from active enhancers lying within protein-coding genes [20]. These unidirectional intragenic lncRNAs (named meRNAs) use the splicing and polyadenylation signals of the protein-coding hosts [20] to produce stable, polyadenylated lncRNAs. Like their intragenic counterparts, intergenic enhancers are also known to be associated with the transcription of both bidirectional as well as unidirectional noncoding RNA transcripts [17-19]. The earlier reports on the association between intergenic lncRNA expression and enhanced levels of neighboring protein-coding genes [10,21,22,45] together with this finding led us to hypothesize that a relatively high, yet undetermined, proportion of intergenic lncRNAs may similarly originate from active intergenic enhancers.

Here we stringently annotated a set of intergenic lncRNAs and, using recent nanoCAGE technology, accurately identified their transcriptional start sites. This enabled us to subclassify these lncRNAs as arising from promoters (plncRNAs, enriched for H3K4me3 over H3K4me1) or enhancers (elncRNAs, enriched for H3K4me1 over H3K4me3) based on the chromatin signatures of their TIRs. Unexpectedly, we found that approximately half of these intergenic lncRNAs are transcribed from enhancers rather than promoters. Importantly, it is the ratio between H3K4me1 and H3K4me3 chromatin marks that distinguishes enhancer- from promoter-like sequences: most TIRs have both chromatin marks (Figure 1) and three-quarters of the TIRs associated with the intergenic enhancers studied here were (to some extent) modified by H3K4me3. We propose that to subclassify intergenic lncRNAs in combination with high resolution maps of H3K4me1 and H3K4me3, the data derived from the same sample that are required to accurately distinguish between these two classes of lncRNAs are (i) high depth poly(A)-selected RNA sequencing, which is needed to faithfully build long transcript models, (ii) nanoCAGE sequencing, which is required to accurately define the 5′ ends of transcripts, and (iii) DNase 1 hypersensitive site sequencing, which allows the identification of regions of open chromatin that often harbor functional *cis*-elements.

Our studies show that plncRNAs and elncRNAs differ substantially in several properties, a distinction that should now facilitate the design of targeted experiments seeking a mechanistic understanding of the biological role of lncRNA transcription, a key question in the field. Despite their different origins, plncRNAs and elncRNAs differ substantially in their levels and tissue or cell expression profiles and in their sequence conservation during evolution. However, they are indistinguishable in terms of their lengths or their numbers of exons or their transcriptional directionality. Previous work has shown that expression of intergenic lncRNAs in general is associated with enhanced expression of their neighboring protein-coding genes, both through genome-wide [10,21,22] and locus-specific analyses [22,23]. Here we show that this is a feature specific to elncRNAs and not plncRNAs.

The clear difference in the selective pressures that have acted on elncRNA and plncRNA TIRs or loci suggests that, if functional, transcription (or transcripts) of the two classes of lncRNAs may have distinct roles. Importantly, the neutral, or near-neutral, evolution of elncRNAs and the evidence of constraint found for plncRNAs imply that the two lncRNA classes are distinct: a locus will rarely be an elncRNA in one tissue and a plncRNA in another. Enhancer-like lncRNA TIRs and sequences are poorly conserved during mammalian evolution, which is entirely consistent with previous reports of rapid turnover of DNA sequence at enhancers during evolution [47,48]. This might suggest that similarly to their intragenic counterparts [20], promoter directionality of intergenic elncRNAs may derive from *cis*-acting signals found in their genomic vicinity and does not reflect global preservation of functional motifs within elncRNA transcripts. The distributions of splice sites and polyadenylation signals around a transcription start site have recently been shown to be important in determining the directionality and stability of extended RNA transcripts [49]. Due to the relatively small number of lncRNAs annotated in this experiment, our data are insufficient to address this issue for erythroblast lncRNA loci.

By contrast, plncRNA promoters and the associated exons in their processed RNAs appear to have evolved under selective constraint, accumulating significantly fewer substitutions than neutral sequence, a signature of potential function. Constraint on plncRNA transcribed sequence occurs predominantly in the exons, indicating that it is the mature, spliced form, rather than the precursor molecule (or more simply the act of transcription) that mediates any plncRNA function. It should be borne in mind that plncRNA constraint is modest, only approximately 20% or approximately 5% of deleterious substitutions are predicted to have been purged from their TIRs or exons, respectively. The latter constraint suggests that either a small fraction of plncRNAs or a relatively small portion of their sequence has conserved (potentially functional) roles [4,6-8].

## Conclusions

In summary, we have shown that the regulatory elements at the origin of intergenic lncRNAs, defined based on chromatin status, allow the distinction between two classes of transcripts that may have different biological functions. This categorization of previously indistinguishable lncRNAs will allow more specific genome-wide investigation of their properties, accelerating our understanding of their potential biological roles and the molecular mechanisms by which they operate.

## Materials and methods

### Primary cells

Biological material for the preparation of high-throughput sequencing libraries was obtained from primary erythroid cells isolated from spleens of phenylhydrazine-treated mice (C57BL/6) [50] using anti-Ter119 microbeads (Miltenyi Biotec { Bergisch Gladbach, Germany }) as previously described [51]. Total RNA used for qPCR was extracted from colony-forming unit erythroblasts (CFUEs) and terminally differentiated primary erythroid (Ter119^+^) cells isolated from E12.5 mouse fetal livers. To enriched samples, cells were expanded *in vitro* for 4 days in StemPro media (Invitrogen, Carslbad, CA, USA) supplemented with erythropoietin (1 U/ml), stem cell factor (50 ng/ml) and dexamethasone (1 μM) at 37°C, 5% CO_2_, followed by magnetic-activated cell sorting depletion of Ter119^+^ cells and FACS sorting of Ter119^neg^/CD44^hi^ progenitor cells (CFUEs) - adapted from Chen *et al.*[52]. To obtain Ter119^+^ terminally differentiated erythroid cells from the same culture, Ter119^neg^/CD44^hi^ progenitor cells were FACS sorted as above and cultured StemPro media (Invitrogen) supplemented with erythropoietin (5 U/ml) without stem cell factor and dexamethasone for for 44 h (37°C, 5% CO_2_).

### Library preparation

Total RNA was extracted using Tri-reagent, DnaseI treated with Turbo DnaseI (Ambion Carlsbad, CA, USA). Good quality RNA (RIN value >9) was poly(A) selected using the PolyATract mRNA isolation system (Promega, Madison, WI, USA) according to the manufacturer’s instructions. Poly(A) + RNA was further depleted of globin transcripts using GlobinClear (Ambion). Libraries for RNA-Seq were prepared and sequenced (51 bp paired-end reads) using the standard Illumina protocol. Strand-specific NanoCAGE libraries from poly(A)-selected RNA were prepared and sequenced (100 bp paired-end reads) by Source Bioscience. (Nottingham, UK).

Chromatin immunoprecipitation on primary erythoid cells was performed as previously described [20] using the following antibodies: anti-H3K4me1 (Millipore, 07–436, Billerica, MA, USA)) and anti-H3K4me3 (Millipore, 05-745R clone 15-10C-E4). ChIP-Seq libraries were prepared and sequenced (50 bp paired-end reads) using the standard Illumina protocol.

### Read mapping and next generation sequencing data analysis

ChIP-seq and DNAse I hypersensitive site (DHS)-seq data were aligned to the mm9 mouse genome build using bowtie (version 0.12.3) [53,54] with the -m reporting option set to 2. To exclude over-amplified products from these data sets, read pairs that map to the identical genomic location were collapsed into a single representative set of reads. Peaks of DNAse I enrichment were called using Seqmonk (version 0.21.1) [55] using the following parameters: depth cutoff = 20; minimum size = 50 and merge distance = 100. The resulting peak calls were intersected with problematic copy number regions in the mm9 genome using intersectappend.pl and used to generate a MIG database [56] for manual inspection of called peaks. A final set of robust DHS peaks were exported from the MIG database excluding problematic copy number regions with a normalized enrichment value of at least 1.

Globin-depleted poly A-selected RNA sequencing reads were mapped to the mouse genome (mm9) with TopHat [57]. Splice junctions from ENSEMBL 68 [58] were provided to facilitate read mapping across known mouse transcript splice junctions. Transcripts were assembled *de novo* using Cufflinks (version 1.3.0) [43] with parameters --min-frags-per-transfrag 5 -m 150 -s 30 -u. ENSEMBL 68 [58] gene annotations were used as reference.

Prior to analysis of the nanoCAGE data the first 21 bp of the read 1 fastq file was trimmed to remove the nanoCAGE specific primer. The resultant paired-end reads were aligned using TopHat (1.1.4b). Aligned reads were split into forward and reverse strands using Samtools [59]. For visualization in genome browsers the position of the first mapped base on read 1 was then used to generate the density of transcription start site positions in a moving window of 100 bp with a 10 bp increment of movement, to produce wig tracks of signal distribution.

### Annotation of transcriptional initiation regions

We used globin-depleted poly(A) + selected nanoCAGE sequencing reads to annotate genome-wide transcriptional start sites and promoters as described elsewhere [25]. Briefly, we extracted the 5′ end position of each read (hereafter termed transcriptional start site). Transcriptional start sites closer than 20 bp and derived from the same strand were clustered. Clusters within 400 bp of each other and on the same strand were considered to be part of the same TIR. TIRs supported by fewer than 5 nanoCAGE reads were discarded, leaving a set of 64,619 TIRs.

For each transcript assembled using cufflinks, we identified reads supporting any of the 64,619 TIRs that overlapped (by >1 nucleotide) their associated transcribed sequence (exons). We excluded from our analysis TIRs that did not overlap (by >1 nucleotide) a DNAse I hypersensitive site region annotated as described above. We associated 14,689 transcripts to the remaining high confidence 11,131 TIRs. For single exonic transcripts only those with a putative TIR upstream of only one of the possible putative transcriptional starts were considered. For these transcripts the strand was imputed based on the strand information for their respective TIRs. This resulted in the annotation of 11,689 transcripts. We considered transcripts overlapping (>1 nucleotide) a protein-coding gene annotation (ENSEMBL build 68) as intragenic (11,036 transcripts). The protein-coding potential of intergenic transcripts longer than 200 nucleotides was analyzed using CPC [33]. Intergenic transcripts longer than 200 nucleotides with no protein-coding potential according to CPC ('noncoding’) and no overlap with pseudogene annotations (ENSEMBL build 68) were annotated as lncRNAs (391 lncRNAs). The relative enrichment of H3K4me1 and H3K4me3 around each TIR or DHS was calculated as previously described using comparably sized libraries [20] and stored in a MIG database. The sorted distribution of high-throughput sequencing data over TIRs (Figure 1) and all DHSs (Additional file 5) was generated using the in-house perl script Hotpile.pl and visualized in R using the gplots library. The cumulative distribution of high-throughput sequencing data over all of the enhancer and promoter populations (Figure 2B,C) and elncRNA and plncRNA TIRs (Figure 2D,E) were generated using the in-house perl script Quantpile.pl and displayed in Microsoft Excel. The strand-specific poly(A)- transcriptional data were split into plus and minus strand transcription using Samtools. The strand-specific enrichment of aligned reads around each class of TIR was determined using the in-house PERL script SRP.pl. The enrichment of poly(A) + aligned reads was determined as for the poly(A)- without splitting the data into forward and reverse strand as this RNA-seq library was non-directional.

### Nucleotide constraint

Nucleotide constraint between mouse and rat locus, exon, intron or TIR was estimated as described previously [8]. Pairwise substitution rates between mouse and rat or human genomic regions were estimated using BASEML from the PAML package with the REV substitution model [60]. The substitution rate of the region of interest was compared to the rate observed for non-overlapping adjacent (<500 kb) ancestral repeats (inserted before the primate and rodent split) with similar G + C content [8].

### Gene expression

Mouse protein-coding transcript annotations were downloaded from ENSEMBL (build 68). Cufflinks (default parameters) was used to estimate the expression of protein-coding genes and lncRNAs using the poly(A)-selected RNA sequencing from intermediate erythroblast (Ter119+) and mapped reads from mouse ENCODE project that were publicly available [44] (Additional file 12).

We estimated the median expression and tissue specificity across all mouse tissue/cell lines (Additional file 12). We calculated tissue specificity (T_*S*_) values for each tissue and each locus. T_*S*_ is defined as the fractional expression of a locus in one tissue relative to the sum of its expression in all tissues. The maximum T_*S*_ value (maxT_*S*_) for a locus thus provides an indicator of tissue specificity, with higher values reflecting more tissue-specific expression [61].

### Association between intergenic lncRNA and neighboring gene expression

The Genome Association Tool (GAT) [62] was used to assess the significance between lncRNA and expression of their neighboring protein-coding genes. For each protein-coding gene we defined its territory as the genomic region containing all nucleotides that are closer to the gene than they are to its most proximal up- and downstream protein-coding genes. We estimated the enrichment in lncRNA transcription in the territories of genes that were expressed more highly (2×, 5× and 10×) in intermediate erythroblasts to what would be expected based on random placement of intervals with a similar size across the intergenic mouse genome.

We downloaded total RNA sequencing reads [28] from the Gene Expression Omnibus (GEO) [63] and mapped the reads to the mouse reference genome (mm9) as described above. We masked the α-globin locus because it accounts for a disproportionate fraction of intermediate erythroblast RNA. The number of mapped reads in CFUEs (colony-forming unit erythroid) and BFUEs (burst-forming uniterythroid) was down sampled to the size of the Ter119+ library, which had the lowest number of mapped reads, by randomly sampling. To estimate normalized gene expression across the three stages of erythropoiesis, we defined a set of constitutive exons for protein-coding gene annotations (ENSEMBL 68) and lncRNAs and quantified the number of sequencing reads mapping to constitutively expressed regions of these transcripts in each of this regions. To allow the comparison of gene expression between species, read counts were normalized using TMM (edgeR package) [64]. Briefly, to estimate the normalized library size for each species, it was assumed that 60% of expressed genes were transcribed at similar levels in the two species. The normalized library sizes were used to calculate the expression level (as total FPKM) of each gene across the three stages of erythropoiesis. Each locus was paired with its genomically closest protein-coding gene. Only pairs where both loci were expressed in at least one erythropoietic stage were considered.

Protein-coding genes and their closest neighboring five elncRNAs and five plncRNAs were selected from the total number of identified candidates in each class (124 and 115, respectively). RNA was extracted from primary mouse CFUEs and terminally differentiated (Ter119^+^) cells using TriReagent (Sigma, St. Louis, MO, USA). RNA was DNase treated (Ambion), and reverse transcribed using SuperScript III (Invitrogen). Expression of each elncRNA/plncRNA and its nearest protein-coding gene was analyzed by qPCR using SYBR Green (Applied Biosystems, Carlsbad, CA, USA). Expression analysis was performed in three independent biological replicates, and normalized to expression of Rn18s (ribosomal subunit 18 s). All reactions were performed in triplicate on each template using the StepOnePlus thermocycler (Applied Biosystems). The sequences of the primers used are available in Additional file 17.

### Data availability

All data used for this analysis are accessible through GEO accession number GSE49460.

## Abbreviations

bp: Base pair; CFUE: Colony forming unit erythroblast; ChIP-seq: Chromatin immunoprecipitation followed by sequencing; DHS: DNAse I hypersensitive site; elncRNA: Intergenic enhancer-associated lncRNA; eRNA: Enhancer-associated RNA; FPKM: Fragments per kilobase of sequence per million reads; GEO: Gene Expression Omnibus; H3K4me1: Monomethylation of lysine 4 of histone H3; H3K4me3: Trimethylation of lysine 4 of histone H3; H3K27ac: Acetylation of lysine 27 of histone 3; lncRNA: Long noncoding RNA; meRNA: Enhancer-associated intragenic lncRNA; PCR: Polymerase chain reaction; plncRNA: Promoter-associated lncRNA; qPCR: Quantitative PCR; TIR: Transcriptional initiation region.

## Competing interests

The authors declare that they have no competing financial interests.

## Authors’ contributions

ACM, JH, MSK, DRH and CPP designed the experiment; MSK isolated the erythroblasts and prepared samples for the RNA-seq and ChIP-seq experiments; ACM and JH processed and mapped the reads; ACM and JH performed genome-wide analysis; BG isolated erythroid populations at specific stages during differentiation and performed qPCR validations; ACM, JH, BG, DRH and CPP discussed and contextualized the results; ACM, JH, BG, DRH and CPP wrote the manuscript; DRH and CPP supervised the study. All authors read and approved the final manuscript.

## Supplementary Material

Additional file 1 Genomic coordinates of intermediate erythroblast expressed transcripts. Click here for file

Additional file 2 Genomic coordinates of transcriptional initiation regions (TIRs) associated with intermediate erythroblast expressed transcripts. Click here for file

Additional file 3 Relationship between TIRs and transcripts. Click here for file

Additional file 4 **The number of tags supporting transcription initiation correlates with its expression.** The logarithm of the number of reads supporting a given transcriptional initiation region (TIR, x-axis) and the expression (log(FPKM), y-axis) of its associated transcripts are significantly correlated (Pearson correlation R = 0.44, *P* < 2 × 10^-16^). Click here for file

Additional file 5 **Contrasting epigenetic landscapes at transcriptional start sites of promoter or enhancer-associated lncRNAs in mouse intermediate erythroblasts.** **(A)** All detected mouse erythroid DNAse I hypersensitive sites (DHSs) were sorted based on the difference in enrichment of H3K4me1 and H3K4me3. The same sort order was used for all panels displayed here, and levels of H3K4me3 and H3K4me1 are depicted as red and blue triangles, respectively. Each panel shows the distribution of signal in a 4 kb window centered on the middle of each DHS. The enhancer and promoter populations are demarcated by blue and red boxes, respectively. The active chromatin mark H3K27ac and NanoCage mapped sources of transcription are associated with both the enhancer and promoter populations, whereas the signal for the tissue-specific transcription factor Gata1 is predominately associated with the enhancer class as expected. **(B,C)** Chromatin profiles normalized for number of peaks associated with the mouse Ter119+ promoters and enhancers, respectively. **(D,E)** Chromatin profiles normalized for number of peaks associated with the mouse Ter119+ plncRNAs and elncRNAs TIRs, showing their promoter and enhancer profiles, respectively. Color coding indicating each chromatin mark is shown below. Click here for file

Additional file 6 Classification of TIRs. Click here for file

Additional file 7 **Examples of previously annotated meRNAs that were also identified in this study.** High resolution maps of DNAse I (black), H3K4me1 (dark blue), H3K4me3 (light blue), Gata1 (red), nanoCAGE (minus stand, dark green; plus strand, light green) and RNA-seq (orange) across mouse **(A)** *Nprl3* (chr11:32123000 to 32175999), **(B)** *Ccdc88c* (chr12:102134000 to 102272999), **(C)** *Inpp5d* (chr1:89519000 to 89642999), **(D)** *Dnttip1* (chr11:32045920 to 32245919) and **(E)** *Hagh* (chr17:24962000 to 25015999). Grey represents the regions previously annotated as enhancer-associated alternative first exons [20]. Click here for file

Additional file 8 Genomic coordinates of intermediate erythroblast expressed elncRNAs. Click here for file

Additional file 9 Genomic coordinates of intermediate erythroblast expressed plncRNAs. Click here for file

Additional file 10 **Distance between pairs of intermediate erythroblast expressed transcripts.** Distance in kilobase pairs between the transcriptional start sites of pairs of genomically neighboring protein-coding gene transcripts (grey); elncRNAs and neighboring protein coding genes (green); and, plncRNAs and neighboring protein-coding genes (blue). ***P < 0.001; NS, not significant. Click here for file

Additional file 11 **Cumulative poly(A) depleted RNA sequencing reads around transcriptional start sites.** Total number of poly(A)- RNA sequencing reads (y-axis) associated with the transcriptional start sites of protein-coding gene, plncRNA and elncRNA meta-genes’ transcriptional start sites (±200 bp, x-axis) originating from the sense (blue) and antisense(red) direction. Arrow indicates direction of transcription. Click here for file

Additional file 12 Intergenic lncRNA expression across different mouse tissues and cell types, including intermediate erythroblasts (Ter119+). Click here for file

Additional file 13 **Exons and introns of elncRNAs have not been selectively constrained during rodent evolution.** Mouse-rat nucleotide substitution rates for elncRNA exons, introns and neighboring putatively neutrally evolving sequence (ancestral repeats, ARs) are shown. NS, not significant. Click here for file

Additional file 14 **plncRNA but not elncRNA loci are selectively constrained over mammalian evolution.** **(A)** Mouse-human nucleotide substitution rates for protein-coding (PC) transcriptional initiation regions (TIRs), lncRNA TIRs and neighboring putatively neutrally evolving sequence (ancestral repeats, ARs). **(B)** Mouse-human nucleotide substitution rates for transcribed loci of protein-coding genes (PC) and lncRNAs and for ARs. **(C)** Mouse-human nucleotide substitution rates for elncRNA and plncRNA TIRs. **(D)** Mouse-human nucleotide substitution rates for elncRNA and plncRNA transcribed loci. ***P < 0.001; **P < 0.01; *P < 0.05; NS, not significant. Click here for file

Additional file 15 **Expression of elncRNAs, plncRNAs and protein-coding genes through three stages of erythropoiesis.** Percentage of loci with detectable expression in one (white), two (grey) or three (black) erythropoiesis stages; data from [26]. Click here for file

Additional file 16 Fold-change in expression. Click here for file

Additional file 17 Primer sequence. Click here for file
