# Supplementary figures and images for: Chromatin signatures at transcriptional start sites separate two equally populated yet distinct classes of intergenic long noncoding RNAs
Source: Genome Biol. 2013 Nov 29;14(11):R131. doi: 10.1186/gb-2013-14-11-r131 (PMC4054604; doi:10.1186/gb-2013-14-11-r131)

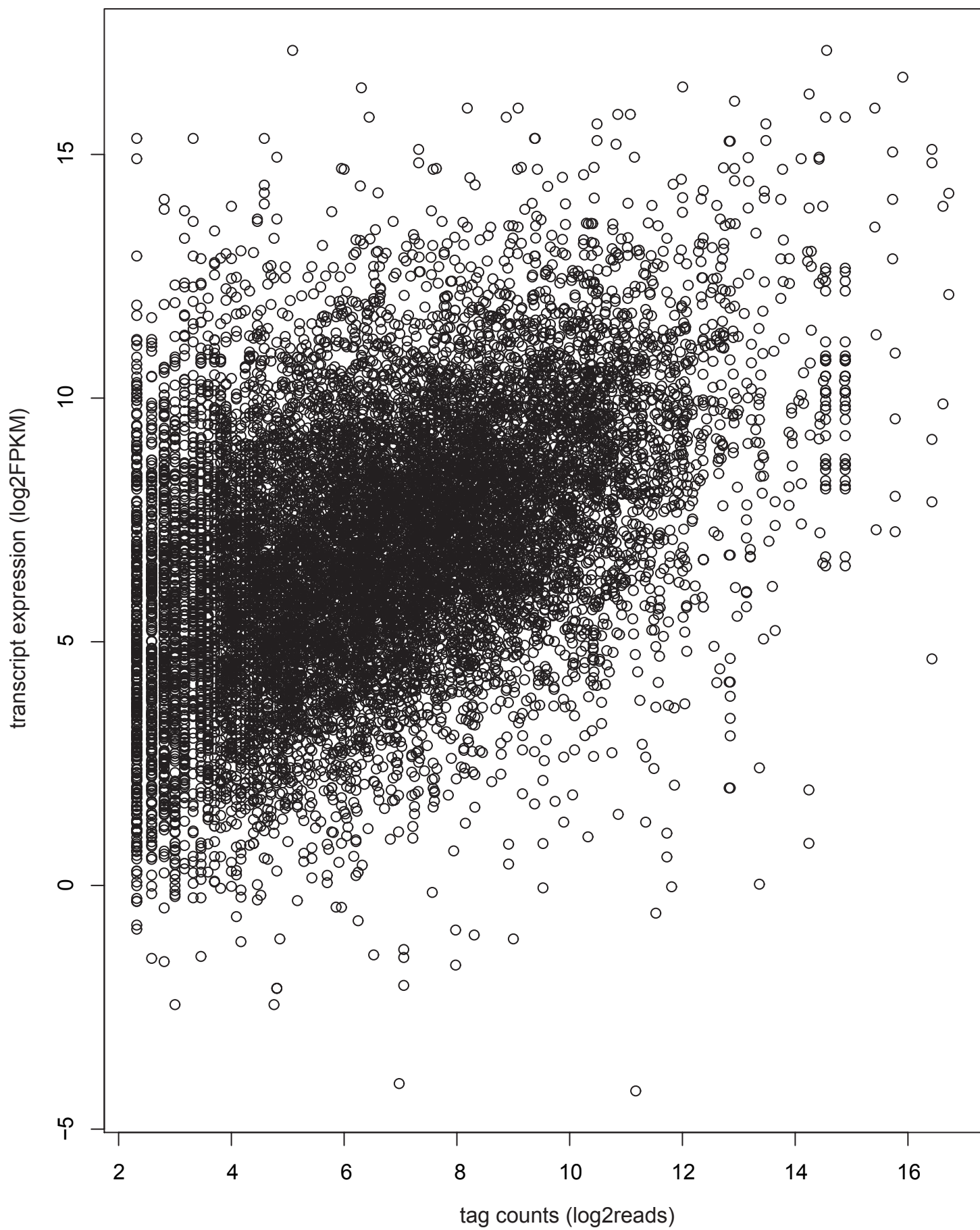

Supplement: Additional file 4 — The number of tags supporting transcription initiation correlates with its expression. The logarithm of the number of reads supporting a given transcriptional initiation region (TIR, x-axis) and the expression (log(FPKM), y-axis) of its associated transcripts are significantly correlated (Pearson correlation R = 0.44, P < 2 × 10^-16^). [file gb-2013-14-11-r131-S4.pdf]

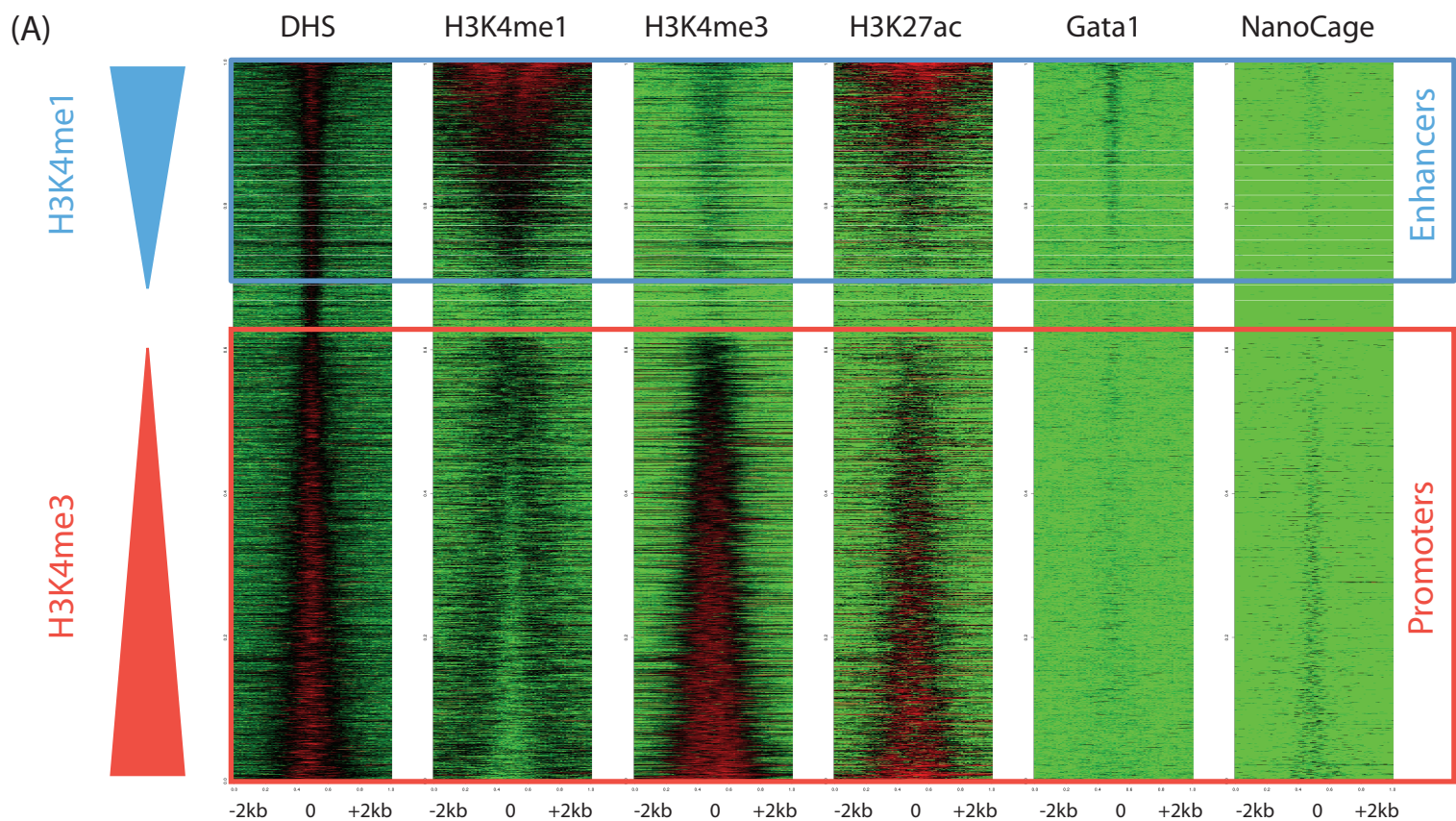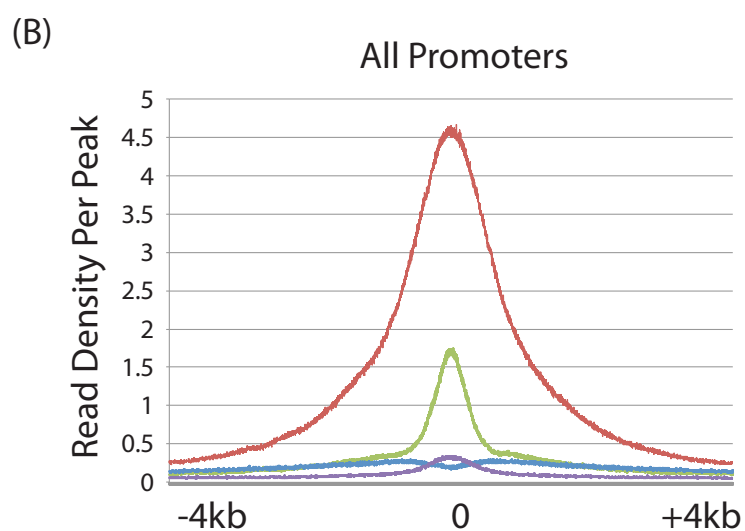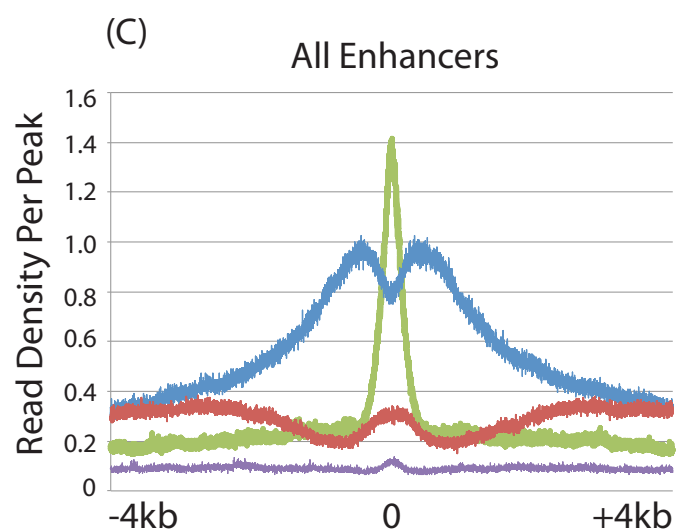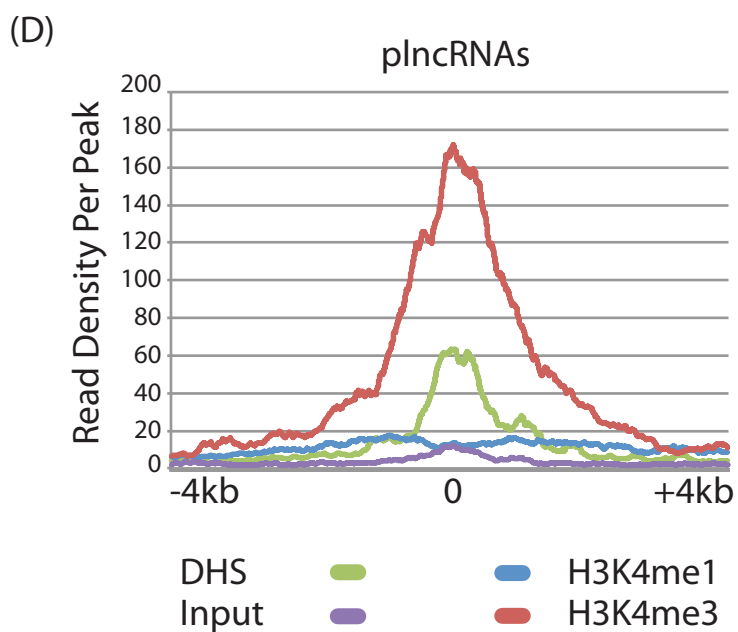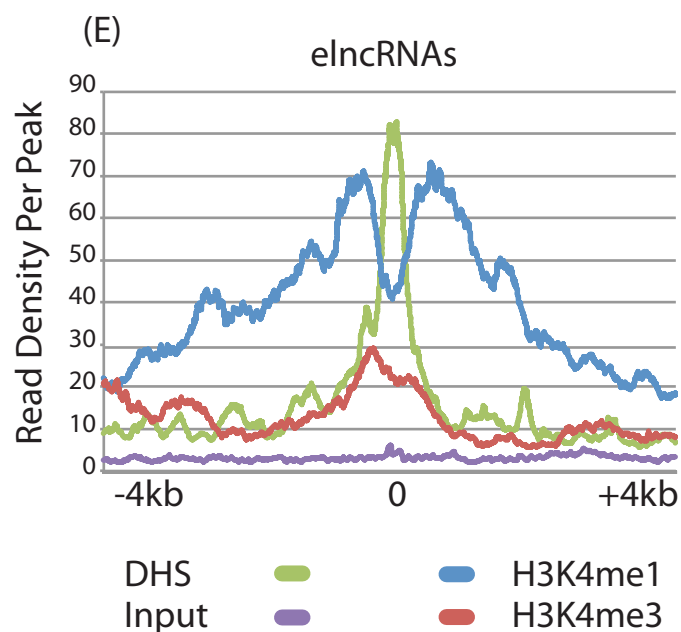

Supplement: Additional file 5 — Contrasting epigenetic landscapes at transcriptional start sites of promoter or enhancer-associated lncRNAs in mouse intermediate erythroblasts. (A) All detected mouse erythroid DNAse I hypersensitive sites (DHSs) were sorted based on the difference in enrichment of H3K4me1 and H3K4me3. The same sort order was used for all panels displayed here, and levels of H3K4me3 and H3K4me1 are depicted as red and blue triangles, respectively. Each panel shows the distribution of signal in a 4 kb window centered on the middle of each DHS. The enhancer and promoter populations are demarcated by blue and red boxes, respectively. The active chromatin mark H3K27ac and NanoCage mapped sources of transcription are associated with both the enhancer and promoter populations, whereas the signal for the tissue-specific transcription factor Gata1 is predominately associated with the enhancer class as expected. (B,C) Chromatin profiles normalized for number of peaks associated with the mouse Ter119+ promoters and enhancers, respectively. (D,E) Chromatin profiles normalized for number of peaks associated with the mouse Ter119+ plncRNAs and elncRNAs TIRs, showing their promoter and enhancer profiles, respectively. Color coding indicating each chromatin mark is shown below. [file gb-2013-14-11-r131-S5.pdf]

**A**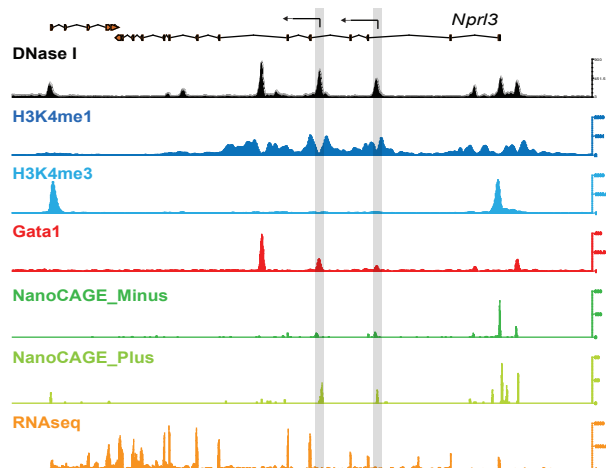**B**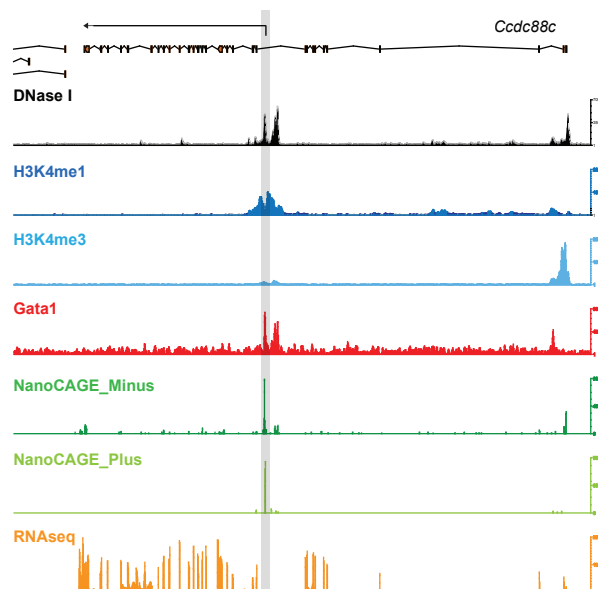**C**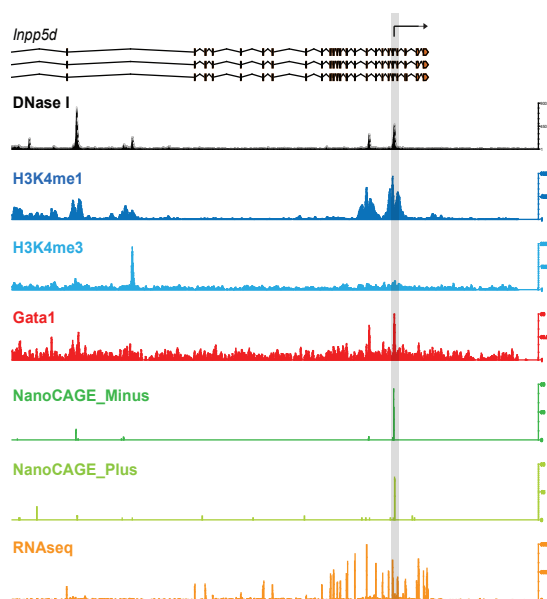**D**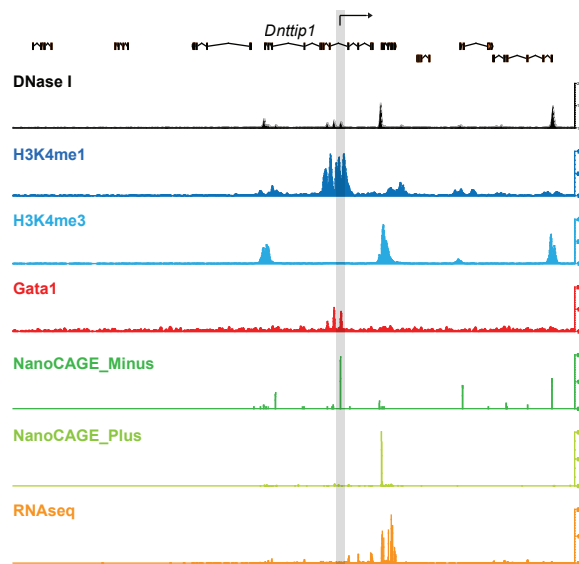**E**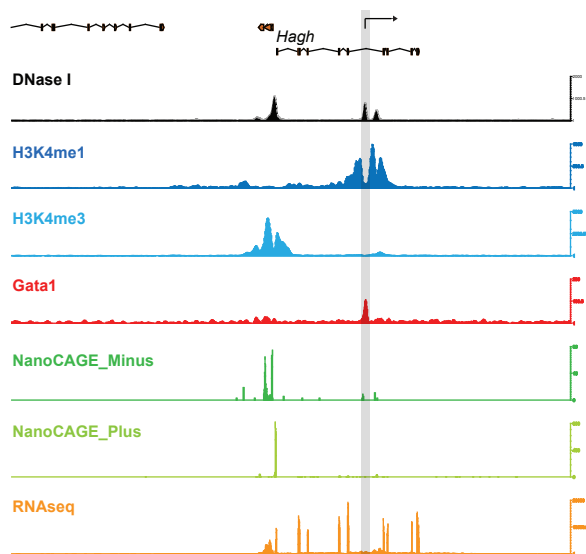

Supplement: Additional file 7 — Examples of previously annotated meRNAs that were also identified in this study. High resolution maps of DNAse I (black), H3K4me1 (dark blue), H3K4me3 (light blue), Gata1 (red), nanoCAGE (minus stand, dark green; plus strand, light green) and RNA-seq (orange) across mouse (A) Nprl3 (chr11:32123000 to 32175999), (B) Ccdc88c (chr12:102134000 to 102272999), (C) Inpp5d (chr1:89519000 to 89642999), (D) Dnttip1 (chr11:32045920 to 32245919) and (E) Hagh (chr17:24962000 to 25015999). Grey represents the regions previously annotated as enhancer-associated alternative first exons [20]. [file gb-2013-14-11-r131-S7.pdf]

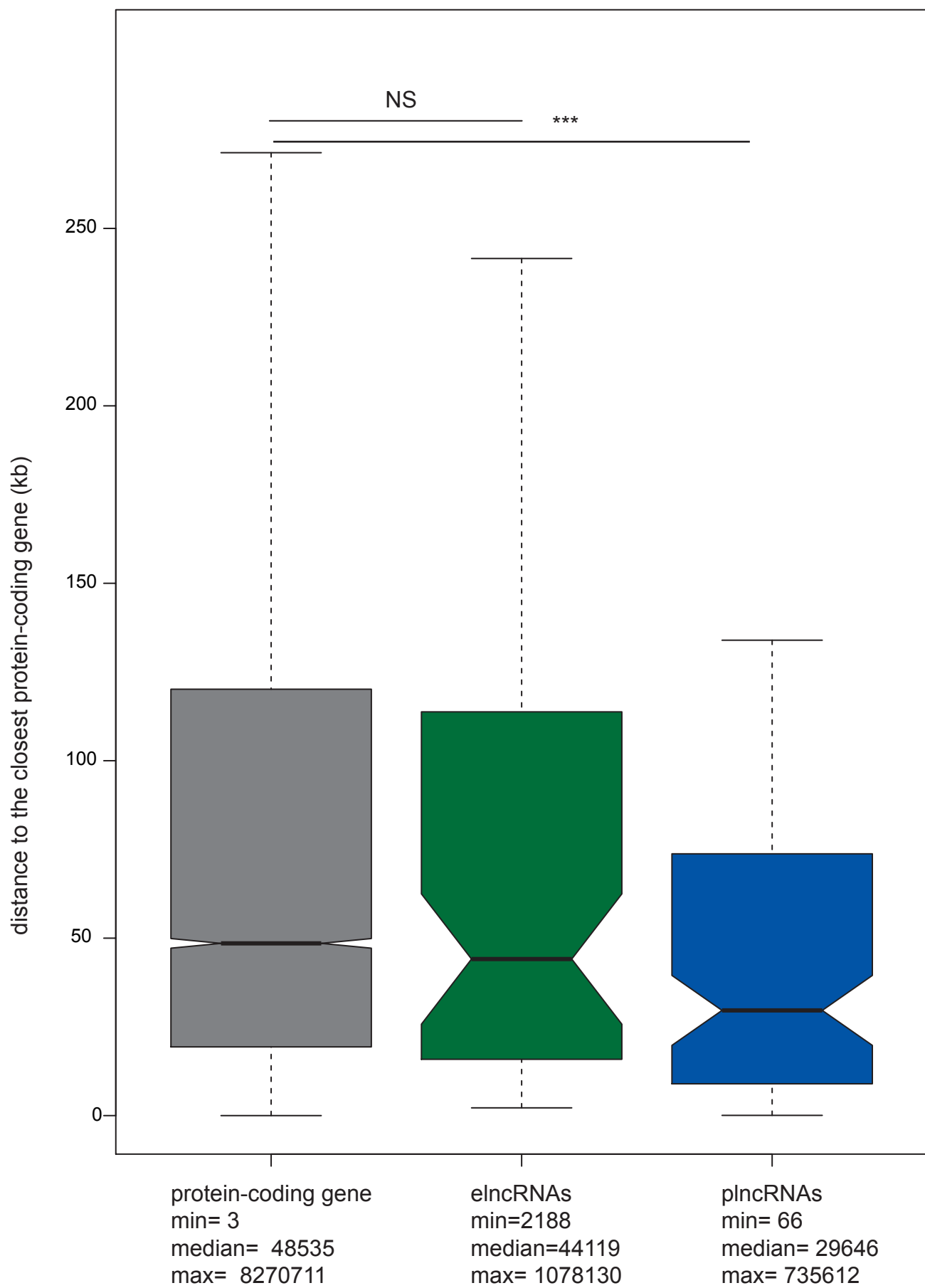

Supplement: Additional file 10 — Distance between pairs of intermediate erythroblast expressed transcripts. Distance in kilobase pairs between the transcriptional start sites of pairs of genomically neighboring protein-coding gene transcripts (grey); elncRNAs and neighboring protein coding genes (green); and, plncRNAs and neighboring protein-coding genes (blue). ***P < 0.001; NS, not significant. [file gb-2013-14-11-r131-S10.pdf]

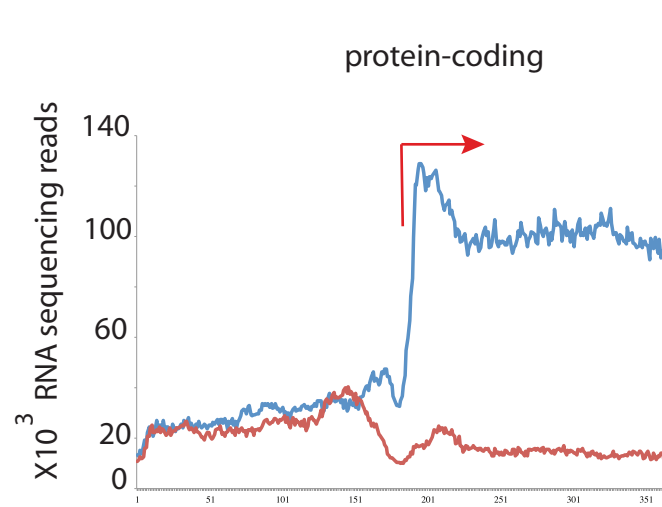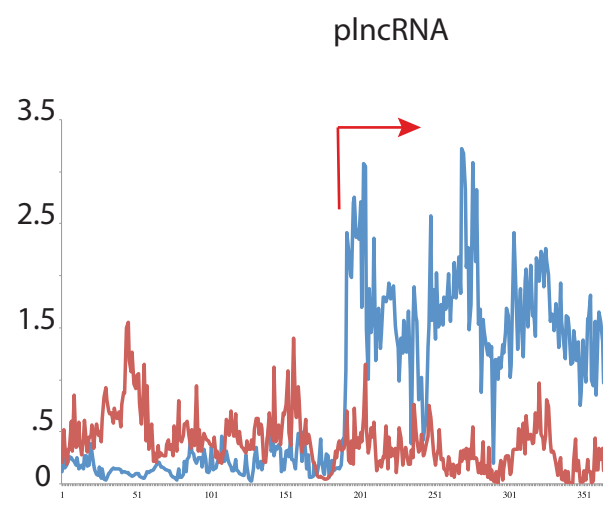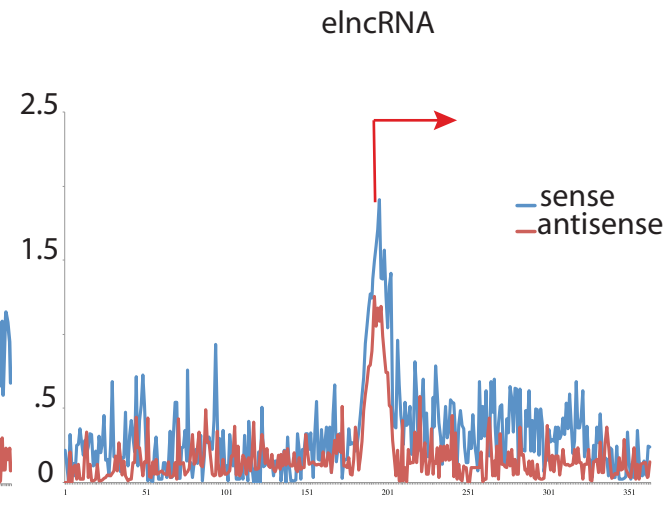

Supplement: Additional file 11 — Cumulative poly(A) depleted RNA sequencing reads around transcriptional start sites. Total number of poly(A)- RNA sequencing reads (y-axis) associated with the transcriptional start sites of protein-coding gene, plncRNA and elncRNA meta-genes’ transcriptional start sites (±200 bp, x-axis) originating from the sense (blue) and antisense(red) direction. Arrow indicates direction of transcription. [file gb-2013-14-11-r131-S11.pdf]

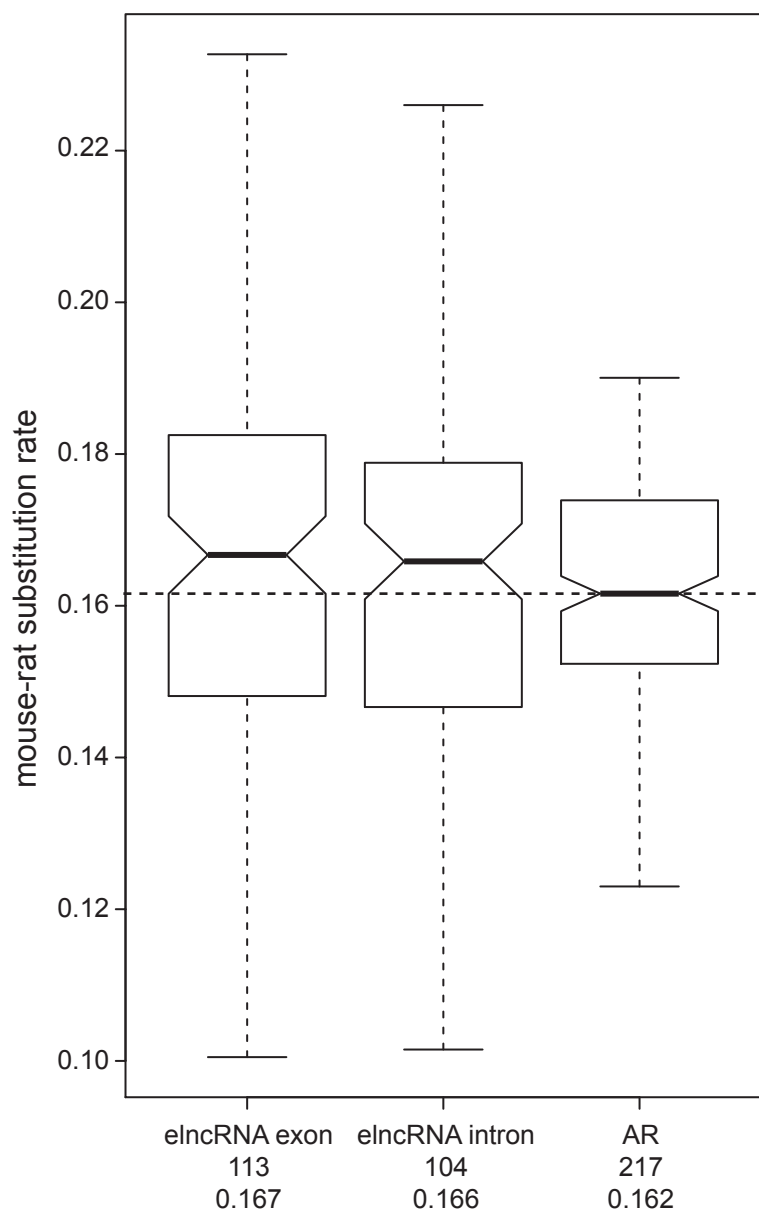

Supplement: Additional file 13 — Exons and introns of elncRNAs have not been selectively constrained during rodent evolution. Mouse-rat nucleotide substitution rates for elncRNA exons, introns and neighboring putatively neutrally evolving sequence (ancestral repeats, ARs) are shown. NS, not significant. [file gb-2013-14-11-r131-S13.pdf]

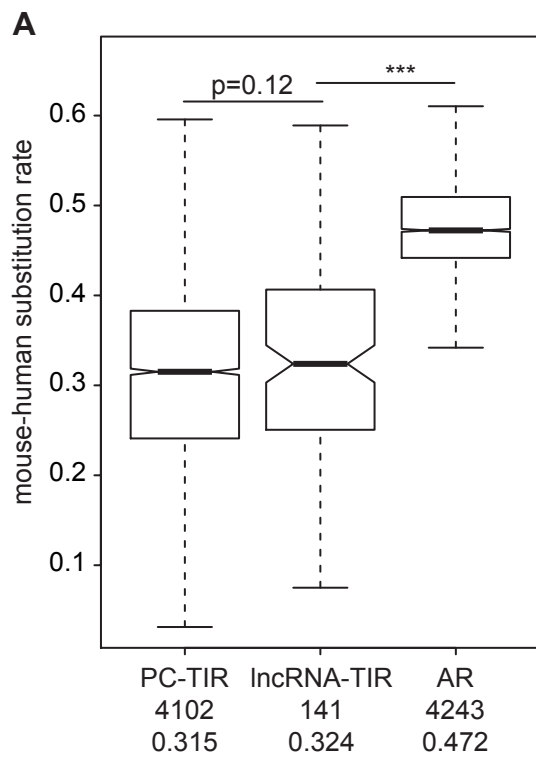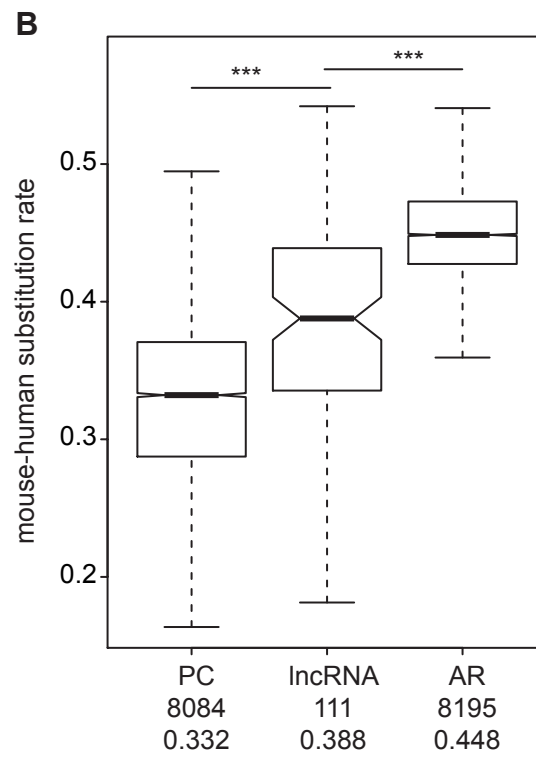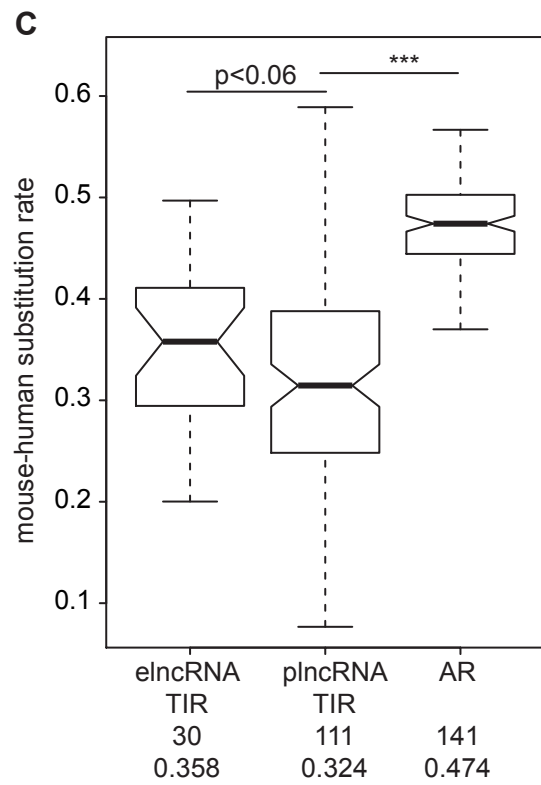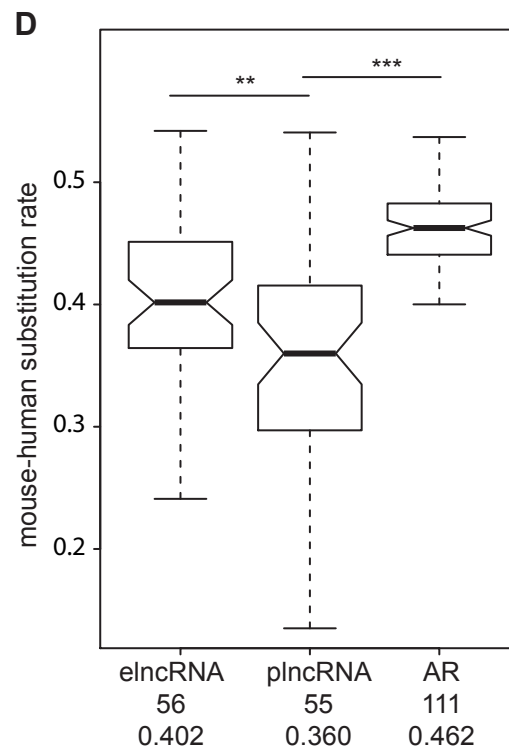

Supplement: Additional file 14 — plncRNA but not elncRNA loci are selectively constrained over mammalian evolution. (A) Mouse-human nucleotide substitution rates for protein-coding (PC) transcriptional initiation regions (TIRs), lncRNA TIRs and neighboring putatively neutrally evolving sequence (ancestral repeats, ARs). (B) Mouse-human nucleotide substitution rates for transcribed loci of protein-coding genes (PC) and lncRNAs and for ARs. (C) Mouse-human nucleotide substitution rates for elncRNA and plncRNA TIRs. (D) Mouse-human nucleotide substitution rates for elncRNA and plncRNA transcribed loci. ***P < 0.001; **P < 0.01; *P < 0.05; NS, not significant. [file gb-2013-14-11-r131-S14.pdf]

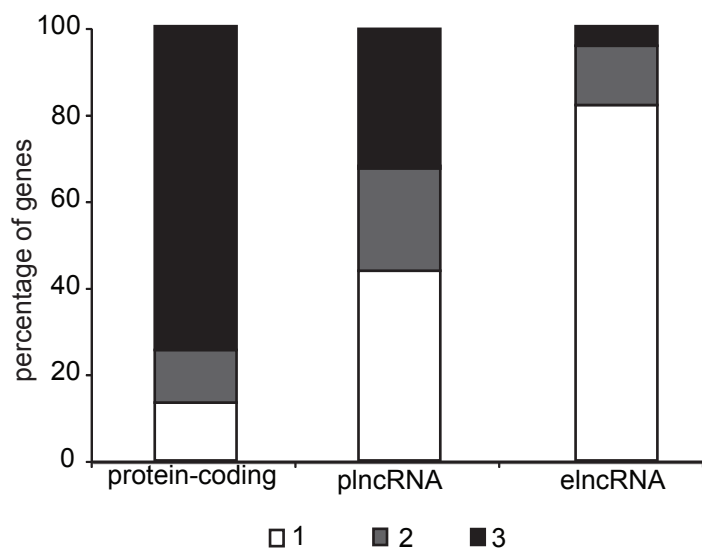

Supplement: Additional file 15 — Expression of elncRNAs, plncRNAs and protein-coding genes through three stages of erythropoiesis. Percentage of loci with detectable expression in one (white), two (grey) or three (black) erythropoiesis stages; data from [26]. [file gb-2013-14-11-r131-S15.pdf]
