# Supplementary material for: Chromatin signatures at transcriptional start sites separate two equally populated yet distinct classes of intergenic long noncoding RNAs
Source: Genome Biol. 2013 Nov 29;14(11):R131. doi: 10.1186/gb-2013-14-11-r131 (PMC4054604; doi:10.1186/gb-2013-14-11-r131)
Supplement: Additional file 17 — Primer sequence. [file gb-2013-14-11-r131-S17.pdf]

elncRNA		
Location	Forward (5' - 3')	Reverse (5' - 3')
chr7.6727.1	ATTGGTCTCTTGGCACCTTGG	GAGACCCTGTGCTCTTTGTCC
chr1.6716.1	GACTGCGCTCTCTCATAGGC	TACGGTCTGTACACGGTGGG
chr17.2833.1	AGAAGTCGCCCCTGTCTGTT	GGGGTGATGTGAGGGTTCCA
chr12.8310.1	GGTTCGCAGGCTCACACTAC	GCAGGTGAGAAGGCTGGTTC
chr3.344.1	CCACGGGCAACTGAAGCTAAT	TGTGGCTGGCATCCAATGAG

Nearest protein-coding gene		
Gene	Forward (5' - 3')	Reverse (5' - 3')
Hbb-y	TGGCCTGTGGAGTAAGGTCAA	GAAGCAGAGGACAAGTTCCCA
Dyrk3	CCCACCCTATTCGGACACATT	TGAAACAGTTGTTCCACCTTCAT
Pim1	CTGGAGTCGCAGTACCAGG	CAGTTCTCCCCAATCGGAAATC
Eif5	AAATCAGTGACCATGCAAAAGGT	TGCCTCAGCCACAATTTCTTTA
Car2	TCCCACCACTGGGGATACAG	CTCTTGGACGCAGCTTTATCATA

plncRNA		
Location	Forward (5' - 3')	Reverse (5' - 3')
chr1.7191.1	AGTCTGGCAGGTTAGTGTGGA	GGTGTCCCTCAGCAACCAAAT
chr6.2692.1	GCTCGGAAGTTTCAGCCCTC	GTGCGTTTCACAAGGCGTTC
chr2.6240.1	TTGACCATCTTCGCCTCCCT	CGACTTGCAGTGTGGGTCTC
chr9.5387.1	GGGCCCGATGTGGATAACAG	TGAGCTCACCTCTCTGCTGG
chr13.2692.1	TAGCTGCGACCTACGGTGAG	CTCCGGCTGCAAGCAATACA

Nearest protein-coding gene		
Gene	Forward (5' - 3')	Reverse (5' - 3')
Btg2	ATGAGCCACGGGAAGAGAA	GCCCTACTGAAAACCTTGAGTC
Jhdm1d	GGCAAACCAGTTAAATCTCAGGG	AGGTTAGAAGGAGTTCGGACAT
Hnrnpa3	GAGGAGGGCCATGATCCAAAG	GTCTGTAAGTGTGCCCCATTT
Mns1	AAGTGGTGCAAAATGAAAACGAC	ATCCTTCAGACTCTCGTGCTT
Nup153	CGGTGAAGCCTTACCAACAGG	GTTCTCATCCGCATAAATCGCA
